# The duration of interventricular septal displacement in patients with precapillary pulmonary hypertension as a potential marker of right ventricular dysfunction and pressure overload. A cardiac magnetic resonance study

**DOI:** 10.1186/1532-429X-16-S1-P240

**Published:** 2014-01-16

**Authors:** Sophia-Anastasia Mouratoglou, Alexandros Kallifatidis, George Giannakoulas, Julia Grapsa, Georgia Pitsiou, Ioannis Stanopoulos, Stavros Hadjimiltiades, Haralambos Karvounis

**Affiliations:** 1 Department of Radiology, St. Luke's Hospital, Thessaloniki, Greece; 2 1st Department of Cardiology, Aristotle University of Thessaloniki, AHEPA University Hospital, Thessaloniki, Greece; 3 Department of Cardiovascular Sciences, Hammersmith Hospital, Imperial College NHS Trust, London, UK; 4 Respiratory Failure Unit, Aristotle University of Thessaloniki, G. Papanikolaou Hospital, Thessaloniki, Greece

## Background

Right ventricular (RV) pressure overload results in interventricular septal displacement (IVSD) towards left ventricle in patients with pulmonary arterial hypertension (PAH). There is however scarce data on the duration of IVSD during cardiac cycle as expressed by curvature duration index (CDi) and its potential role in the evaluation of PAH patients. The aim of our study is to reveal the potential value of CDi as a marker of RV function and pressure overload.

## Methods

All patients underwent cardiac magnetic resonance (CMR, Avanto Siemens 1,5T). A routine set of LV and RV short-axis cines of 6 mm slice thickness, were acquired from base to apex using a breath-hold retrospective ECG-gated balanced steady state free precession (SSFP) sequence. CDi (duration of septal curvature configuration × 100/cardiac cycle duration), left ventricular eccentricity index in end-systole (LVSei) and end-diastole (LVDei), left ventricular end-systolic (LVESarea) and end-diastolic area (LVEDarea), RV end-systolic (RVESarea) and end diastolic area (RVEDarea) were defined in the short-axis view in the level of papillary muscles. Interventricular septal curvature ratio (CR) was defined in the same level, at end-systole. The right ventricular wall thickness (RVWT) was assessed in the anterior segment of RV. Tricuspid annular plane systolic excursion (CMR-TAPSE) was defined in the 4-chamber view. Right ventricular ejection fraction (RVEF) and RV end-systolic volume (RVESV) and end-diastolic volume (RVEDV) were obtained with the use of serial short axis cine-MRI views from base to the apex, according to Simpson's rule.

## Results

Our study included 41 consecutive patients (33 women, mean age 45.6 ± 12.1 years) with precapillary pulmonary hypertension (29 with idiopathic PAH, 7 with PAH associated to congenital heart disease, 2 with PAH associated to connective tissue disease and 3 with chronic thromboembolic pulmonary hypertension). A direct linear correlation between CDi and CMR-TAPSE (r = -0.464, p = 0.02), CR (r = -0.796, p < 0.001), LVSei (r = 0.802, p < 0.001) and LVDei (r = 0.6, p < 0.001), LVESarea (-0.364, p = 0.02), LVEDarea (-0.538, p < 0.001), RVEF (-0.484, p = 0.02), RVESV (0.509, p = 0.01), RVESarea (0.538, p < 0.001), RVEDarea (0.497, p = 0.02), RVWT (0.447, p = 0.004) was observed.

## Conclusions

CDi is a potential non invasive simple marker for the evaluation of RV pressure overload and function in patients with precapillary pulmonary hypertension. Its prognostic significance remains to be established in further studies.

## Funding

Hellenic Cardiological Society.

**Table 1:** 

		Bivariate correlation	
	**mean ± SD**	**r**	**p**

CDi%	68.2 ± 23.5		

CMR-TAPSE (cm)	1.46 ± 0.45	-0.464	0.02

CR	0.61 ± 0.19	-0.796	<0.001

LVSei	1.96 ± 0.84	0.802	<0.001

LVDei	1.47 ± 0.30	0.6	<0.001

LVESarea	13.98 ± 5.35	-0.364	0.02

LVEDarea	27.49 ± 7.44	-0.538	<0.001

RVEF	47.67 ± 11.21	-0.484	0.02

RVESV	64.12 ± 37.44	0.509	0.01

RVESarea	24.29 ± 10.38	0.538	<0.001

RVEDarea	34.86 ± 9.70	0.497	0.02

RVWT	0.65 ± 1.18	0.447	0.004

**Figure 1:**
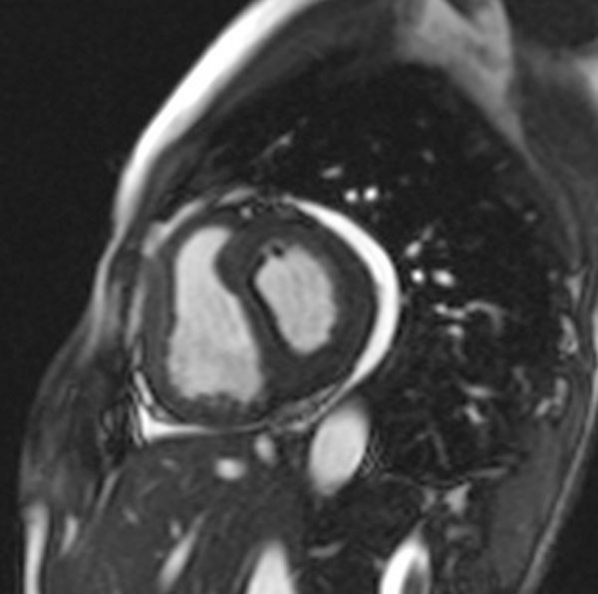
**Basal Short-Axis SSFP image in end-systolic phase showing leftward septal displacement**.

